# Global Use, Adaptation, and Sharing of Massive Open Online Courses for Emergency Health on the OpenWHO Platform: Survey Study

**DOI:** 10.2196/52591

**Published:** 2025-01-10

**Authors:** Jamie Sewan Johnston, Nadine Ann Skinner, Anna Tokar, Elham Arabi, Ngouille Yabsa Ndiaye, Matthew Charles Strehlow, Heini Utunen

**Affiliations:** 1 Stanford Center for Health Education Stanford University Stanford, CA United States; 2 Learning and Capacity Development Unit, Health Emergencies Programme, World Health Organization Geneva Switzerland; 3 Department of Emergency Medicine Stanford University School of Medicine Stanford, CA United States

**Keywords:** MOOCs, online learning, global health education, digital health, health worker training, health emergencies, outbreak, COVID-19

## Abstract

**Background:**

The COVID-19 pandemic demonstrated the global need for accessible content to rapidly train health care workers during health emergencies. The massive open access online course (MOOC) format is a broadly embraced strategy for widespread dissemination of trainings. Yet, barriers associated with technology access, language, and cultural context limit the use of MOOCs, particularly in lower-resource communities. There is tremendous potential for MOOC developers to increase the global scale and contextualization of learning; however, at present, few studies examine the adaptation and sharing of health MOOCs to address these challenges.

**Objective:**

The World Health Organization’s Health Emergencies Programme Learning and Capacity Development Unit and the Stanford Center for Health Education collaborated to survey learners from 4 emergency health MOOCs on the OpenWHO platform to examine differences in course use by World Bank country income classification across three dimensions: (1) how health education MOOCs are used and shared, (2) how health workers adapt MOOC content to meet local training and information needs, and (3) how content adaptations help frontline health workers overcome barriers to using MOOCs.

**Methods:**

This study draws upon two sources of data: (1) course enrollment data collected from the 4 emergency health MOOCs (N=96,395) and (2) survey data collected from learners who participated in at least 1 of the 4 MOOCs (N=926). Descriptive statistics are used to summarize learner characteristics. Differences in enrollment, sharing, and adaptation by country income classification are examined using Pearson chi-square test.

**Results:**

Of the enrollees who indicated their country of residence, half were from lower-middle-income countries (LMICs; 43,168/85,882, 50%) and another 9% (7146/85,882) from low-income countries. The majority of all respondents shared content (819/926, 88%) and used content in official trainings (563/926, 61%). Respondents were more likely to share and use content for trainings in LMICs than in high-income countries (91% vs 81%; *P*=.001). Learners in LMICs also shared content with more people on average compared with high-income country learners although the difference is not statistically significant (9.48 vs 6.73 people; *P*=.084). Compared with learners in high-income countries, learners in LMICs were more likely to adapt materials to distribute via offline formats or technologies, such as WhatsApp or text message (31% vs 8%; *P*<.001); to address cultural, linguistic, or other contextual needs (20% vs 12%; *P*=.076); and to meet local guidelines (20% vs 9%; *P*=.010). Learners in LMICs indicated greater accessibility challenges due to technological and linguistic barriers.

**Conclusions:**

Learners commonly share content from MOOCs about public health emergencies; this is especially true in low-income countries and LMICs. However, content is often adapted and shared via alternative formats. Our findings identify a critical opportunity to improve MOOC design and dramatically scale the impact of MOOCs to better meet diverse global needs.

## Introduction

### Overview

First launched in 2017, the World Health Organization’s (WHO) Health Emergencies Programme’s learning platform, the OpenWHO, uses a massive open access online course (MOOC) format. The platform rapidly expanded during the COVID-19 pandemic, from serving approximately 160,000 learners at the pandemic’s start [1] to serving more than 7.5 million learners in 194 countries and 65 languages by the end of 2022, with 46 courses specifically on COVID-19 and more than 85 additional emergency health course topics [1,2]. The MOOCs on the platform are designed to improve equitable access to emergency health topics according to learners’ needs and preferred languages [3]. Even with the significant expansion in topics and utilization, technical, cultural, and linguistic barriers limit dissemination of the lifesaving knowledge provided through MOOCs. Preliminary research indicated that local health workers and institutions adapt and share digital content to increase knowledge dissemination [4-6]. This study evaluates how MOOC content on the OpenWHO platform is used, shared, and adapted to better understand the ability of MOOCs to reach frontline health workers during public health emergencies.

### Background

Over the past decade, digital education tools have been increasingly incorporated into the training and education of frontline health workers [7-12]. However, the COVID-19 pandemic demonstrated a critical global need to expand easily accessible and trustworthy open-source content for rapid frontline health worker training during health crises. The MOOC format, embraced by the WHO [3,13-16], governments [17,18], and academic institutions worldwide [19,20], is one method for the widespread dissemination of emergency training for frontline health workers.

The MOOC format has been successfully used in public health crises to disseminate information to the public, policy makers, and other stakeholders [21-23] and provide health workers with training and knowledge [15,17,24-30]. While there are different varieties of MOOCs, content is often offered online for learners via readings, wikis, presentation slides, and short videos [31]. MOOCs also typically include learner assessments and interactive components, such as automated quizzes, peer-marked assessments, or online discussion forums, and may be self-paced and asynchronous [31].

While the MOOC format has seen success in public health crises, enrollment in and completion of MOOCs in many low-income and lower-middle-income countries (LICs/LMICs) are disproportionately lower than those in high-income and upper-middle-income countries (HICs/UMICs) [32]. MOOC enrollment and completion vary by region but are substantially lower in countries with limited investment in higher education systems and technology infrastructure [33,34]. Significant technical barriers prevent enrollment and completion of MOOCs in resource-limited and remote settings [6,31-35]. Challenges concerning digital literacy, weak technical support, web access, and broadband cellular coverage persist, limiting the reach and effectiveness of digital health education tools in these regions [27,31,33-38]. Infrastructure barriers can cause challenges when accessing MOOCs, including downloading materials, engaging in interactive elements, and viewing high-resolution videos [31].

There may also be significant cultural and linguistic barriers that prevent MOOC engagement [39-47]. Critics have argued that MOOC content often needs more cultural context for successful curriculum design [31,37,39-48]. Traditional MOOC content has been based on the academic experiences, pedagogical ideas, design concepts, and literature of the Global North [31,39,45,46]. Research has shown that including various learning, assessment, and accreditation activities in MOOC design facilitates learners’ progress in one context while inhibiting learners in other contexts [31,40-47]. In particular, Indigenous groups and knowledge have been noted as lacking in MOOC design and research, which has been seen as a limitation in ensuring the relevance of MOOCs for Indigenous populations [47].

In addition to cultural relevance, MOOCs should also be linguistically relevant [3,16] in languages where learners seek out health information [49] and at literacy levels that can be understood by diverse audiences [50,51]. Communities, especially vulnerable communities, have indicated mistrust in digital health materials that are not culturally and linguistically relevant [39,50,52]. Offering MOOCs in multiple languages successfully enables increased access to diverse regional learners with low levels of previous education [2,43], and cultural and linguistic translations can make MOOCs and digital content more relevant for learners [43] and support their ability to learn the material [53].

Culturally and linguistically relevant content has increased positive health beliefs and behaviors [54], which indicates a need for context-specific adaptations for global MOOCs to support learner needs. Yet, initially, most MOOCs were developed in English with more MOOCs now being created in multiple languages [31]. However, research on infectious disease and health MOOCs has found limited resources in languages other than English [20,55,56]. In addition, when MOOCs have been translated from English into other languages, translations include less accessible word choices, especially for low-literacy populations [43,44]. These translations may also reduce the provided health content’s complexity [56].

Proponents of open-source digital education have argued that learners can adapt and share well-designed materials to overcome access barriers, leading to what has been termed a “learning multiplier effect” that describes the transformation and sharing of web-based resources into adapted formats to suit learners’ needs and local context beyond what can be tracked by platform analytics [6]. Evidence suggests that during health crises, health workers are motivated to enroll in MOOCs to improve practice [57]. The need for trustworthy information also drives health workers to share MOOC content through personal and professional networks [5,6]. Learners have shared MOOC materials via their networks by downloading materials, providing links to resources, and telling others in their networks about the course and course content [5,57]. They also adapted and integrated MOOC resources into formal training, making MOOCs accessible to even larger audiences [4-6]. Adaptations included efforts to overcome technical barriers, such as downloading or making content available via text message [4-6]. Other adaptations included addressing cultural or local context, such as by adding local health regulations or addressing linguistic relevance with local translations [5,6].

### Intervention

The OpenWHO platform is an open access, low-bandwidth requirement online learning platform first launched following the Ebola West Africa outbreak in 2017 [3]. The platform aims to provide lifesaving online knowledge based on the latest WHO technical advice to frontline responders, policy makers, and the public. The platform hosts MOOCs on 33 different infectious diseases in 65 languages aiming to disseminate critical learning materials for any type of health event and outbreak. There are a total of 46 MOOCs related to COVID-19, 5 on Ebola, and a range of courses on different epidemic and pandemic-prone diseases [16].

This study examines 2 MOOCs focused on COVID-19 and 2 on other epidemic and pandemic-prone diseases, Ebola and rabies. These 4 courses were selected because they had large enrollments of health professionals. The 2 COVID-19 courses are “Clinical Management of Patients With COVID-19: General Considerations” and “Clinical Management of Patients With COVID-19: Initial Approach to the Acutely Ill Patient.” The other 2 health emergency courses selected for the study include one of the first courses created for the OpenWHO platform, “Ebola: Clinical Management of Ebola Virus Disease” (developed in response to the 2018 Ebola outbreak in the Équateur Province of the Democratic Republic of the Congo) and a relatively new course, “Rabies and One Health: From Basics to Cross-sectoral Action to Stop Human Rabies Deaths.” The study includes both COVID-19 and other health emergency courses to understand better which course-sharing experiences and adaptations may have been unique to the COVID-19 pandemic context. All of the courses are available in English and 2-4 additional languages. A full description of the MOOCs can be seen in Table 1.

**Table 1:** Course descriptions.

Course title and description	Languages	Date launched	Course duration	Enrolled learners	Course completion
Clinical Management of Patients With COVID-19: General Considerations: this course gives background on the pandemic, discusses facility operations, and addresses COVID-19 pandemic preparedness at all levels of health care provision, including surge planning, infection prevention and control, palliative care, and transfer of the acutely ill patient. It also discusses ethical issues arising during COVID-19 care.	Albanian, Dutch, English, Indonesian, Kazakh, and Macedonian	October 22, 2020	3 hours	38,941	Record of achievement: Earning at least 80% of the maximum points from all graded assignments. Confirmation of participation: Completing at least 80% of the course material.
Clinical Management of Patients With COVID-19: Initial Approach to the Acutely Ill Patient. This course is designed to prepare and support health providers as they provide emergency care to seriously ill COVID-19 patients. The course includes a systematic approach via the WHO/ICRC^a^ Basic Emergency Care course content, covering screening and triage, the integrated interagency triage tool, resuscitation area designation, the WHO medical emergency checklist, the ABCDE approach to the acutely ill patient, and the approach to the patient with breathing difficulty, shock, or altered mental status.	English, Portuguese, Russian, Somali, and Spanish	May 5, 2021	6 hours	24,036	Record of achievement: Earning at least 80% of the maximum points from all graded assignments. Confirmation of participation: Completing at least 80% of the course material.
Ebola: Clinical Management of Ebola Virus Disease: this comprehensive intermediate-level course is for clinicians caring for patients with suspected or confirmed Ebola virus disease. The course covers screening and triage, infection prevention and control, laboratory diagnostics, organization and clinical care of patients in the Ebola Treatment Centre, and investigational therapeutic agents.	English, French, and Kiswahili	2018	6 hours	23,803	No certificate available at this time.
Rabies and One Health: From Basics to Cross-Sectoral Action to Stop Human Rabies Deaths: this course provides a general introduction to rabies, and the One Health approach currently taken to prevent it including information on the “Zero by 30” rabies elimination strategy, preventing rabies in people and dogs, awareness and community empowerment, and diagnosis and surveillance. It targets both a general audience and those who would like to learn more about rabies and the pathway to eliminating this disease.	English, French, and Russian	September 2021	3 hours	9615	Record of achievement: Earning at least 80% of the maximum points from all graded assignments.

^a^WHO/ICRC: World Health Organization/International Committee of the Red Cross.

### Objectives

We surveyed the learners from the 4 emergency health MOOCs to understand current trends in sharing and adapting these courses by learners on the OpenWHO platform. This study analyzes the survey responses to examine differences in course use by World Bank country income classification across the following dimensions: (1) how health education MOOCs are being used and shared by learners, (2) how health workers adapt global health education MOOC content to meet local health education training and information needs, and (3) how content adaptations can help frontline health workers overcome the barriers to using health education MOOCs, especially in LICs/LMICs.

## Methods

The study leverages 2 sources of data. The first source is course enrollment data collected from the start of each course through the end of March 2022 (N=96,395) from the 4 MOOCs on the OpenWHO platform. The second data source is surveys of learners who participated in at least 1 of the 4 MOOCs (N=926) during this same time period.

### Enrollment Data

The enrollment data describe the demand and the ability to access the 4 MOOCs. The data were collected from learners at the time they registered to enroll in the MOOCs. Enrollment data included information on learners’ country of residence, gender, institutional affiliation, age range, primary language, enrollment language, and course completion.

### Survey Data

In March 2022, we distributed a survey via email to learners who enrolled in at least 1 of the 4 MOOCs with a link to participate in the survey. The survey was sent in 3 languages: English (all MOOCs had English versions), French (the Ebola and rabies MOOCs had French versions), and Spanish (the COVID-19 “Initial Approach to the Acutely Ill Patient” had a Spanish version).

The 33-question 3-part survey included sections with questions about (1) which MOOCs the learners took and their experiences with the courses, (2) how they shared and adapted course content, and (3) how accessible the learners found course content (see Multimedia Appendix 1 for the full survey). The 7-question section on learners’ experiences with the MOOCs asked questions about course completion, timing, and motivation (see survey Section A in Multimedia Appendix 1). The 11-question section on course sharing and adaptation asked questions about course recommendations, sharing modalities, sharing experiences, modification modalities, and modification experiences (see survey Section Bin Multimedia Appendix 1). The 16-question section on accessibility asked questions about learner characteristics and accessibility of course content for professional and patient populations (see survey Section C in Multimedia Appendix 1).

### Data Analysis

Enrollment and survey data were summarized using descriptive statistics (mean, SD, and response rates). To understand the generalizability of our survey sample to the overall population of enrollees and course completers, we summarized the learner characteristics of enrollees, course completers, and survey respondents. The learner characteristics we examined include course enrollment, geographic region, country income classification, course language, language preference, professional affiliation, age, and gender. Not all enrollees provided responses to registration questions; we included percentages of those for whom we have data on characteristics.

To investigate the differences in course use across learners globally, we summarized survey data by World Bank country income classifications—HICs, UMICs, LICs, and LMICs. We tested differences in course sharing and adaptation by country income classification using Pearson chi-square test, comparing sharing and adaptation patterns in UMICs, LMICs, and LICs with patterns in HICs.

In an attempt to quantify the course reach that cannot be observed in platform analytics, we calculated a lower-bound estimate of sharing by assuming that each individual shared with the lowest number of people in their respective survey category responses (ie, 0 people, 1-10 people, 11-25 people, 26-50 people, and 50 people). We calculated the lower bound number of recipients reached through sharing, as well as the lower bound number of trainees reached through the use of the course content in official training. We used one-way ANOVA to compare differences in the mean number of recipients and training by country income classification. All statistical analyses were performed using Stata SE version 15.

### Ethical Considerations

Informed consent was obtained from all surveyed participants. Approval for this study was obtained from the Stanford University School of Medicine institutional review board (protocol no. 62414). Data were collected anonymously and respondents did not receive compensation.

## Results

### Enrollment Patterns

A total of 96,395 learners enrolled in the 4 MOOCs from each course’s start date until the end of March 2022. Learners could enroll in more than 1 course. Of the enrolled learners who indicated geographic residence when registering for the OpenWHO platform, half indicated that they were from LMICs (43,168/85,882, 50%), and another 9% (7146/85,882) indicated that they were from LICs. For context, roughly 40% of the world’s population resides in LMICs, while roughly 9% reside in LICs [58]. More than one-third of enrolled learners indicated that they were from South Asia (29,001/85,882, 34%), with the subsequent largest distribution of learners from Sub-Saharan Africa (14,058/85,882, 16%). Examining enrolled learners’ demographics finds that 79% (51,479/64,793) indicated that their preferred language for learning was English, and 88% (84,949/96,395) enrolled in an English language MOOC.

A third of enrolled learners indicated they were students (28,308/87,041, 33%), with the next largest enrolled population being health care professionals (23,773/87,041, 27%). Enrolled learners were generally young, with 85% (50,618/59,896) of learners younger than 40 years. In addition, more than half of the enrolled learners identified as male (35,444/65,430, 54%).

### Course Completion Patterns

MOOCs generally have low completion rates, with rates that can be disproportionately lower in LICs/LMICs [31]. These 4 MOOCs have an overall course completion rate of 34% (32,395/96,395), which ranges from 23% (2165/9615) for the Rabies and One Health course to 40% (9376/23,803) for the Ebola: Clinical Management course. Course completion rates were only slightly lower in LICs (2457/7146, 34%) and LMICs (14,942/43,168, 35%) than in UMICs (7207/19,743, 37%) and HICs (6870/15,825, 43%). As shown in Table 2, course completers generally looked similar to the population that enrolled in the MOOCs with few notable differences.

**Table 2:** Learner characteristics by course enrollment, course completion, and survey completion^a^.

Characteristics			Enrolled in course (N=96,395)		Completed course (N=32,935)		Completed survey (N=926)
**Course, n (%)**							
	COVID-19 Acutely Ill Patients	24,036 (25)		7445 (23)		607 (66)	
	COVID-19 General Considerations	38,941 (40)		13,949 (42)		524 (57)	
	Ebola Clinical Management	23,803 (25)		9376 (28)		254 (27)	
	Rabies and One Health	9615 (10)		2165 (7)		283 (31)	
**Geographic region, n (%)**							
	East Asia and Pacific	11,972 (14)		4413 (14)		79 (9)	
	Europe and Central Asia	6693 (8)		2382 (8)		63 (7)	
	Latin America and Caribbean	9927 (12)		3645 (12)		137 (15)	
	Middle East and North Africa	6760 (8)		2328 (7)		63 (7)	
	North America	7471 (9)		3837 (12)		23 (2)	
	South Asia	29,001 (34)		10,469 (33)		229 (25)	
	Sub-Saharan Africa	14,058 (16)		4402 (14)		332 (36)	
	Not specified	10,513		1459		N/A^b^	
**Country income classification, n (%)**							
	Low income	7146 (8)		2457 (8)		171 (18)	
	Lower-middle income	43,168 (50)		14,942 (47)		441 (48)	
	Upper-middle income	19,743 (23)		7207 (23)		200 (22)	
	High income	15,825 (18)		6870 (22)		114 (12)	
	Not specified	10,513		1459		N/A^b^	
**Course language, n (%)**							
	English	84,949 (88)		28,499 (87)		769 (83)	
	French	3864 (4)		1316 (4)		45 (5)	
	Spanish	4993 (5)		2185 (7)		112 (12)	
	Other	2589 (3)		935 (3)		N/A^b^	
**OpenWHO language preference, n (%)**							
	Arabic	1947 (3)		606 (3)		22 (2)	
	Chinese	2225 (3)		792 (4)		20 (2)	
	English	51,479 (79)		17,720 (80)		627 (68)	
	French	2537 (4)		839 (4)		64 (7)	
	Portuguese	589 (1)		131 (1)		7 (1)	
	Russian	454 (1)		153 (1)		2 (0)	
	Spanish	5562 (9)		1902 (9)		138 (15)	
	Other	N/A^b^		N/A^b^		46 (5)	
	Not specified	31,602		10,792		N/A^b^	
**Professional affiliation, n (%)**							
	Government/Ministry of Health	8491 (10)		2719 (9)		51 (6)	
	Health care professional	23,773 (27)		7387 (24)		406 (44)	
	International organization (eg, WHO, UN)	2532 (3)		974 (3)		23 (2)	
	Nongovernmental organization	3546 (4)		1186 (4)		58 (6)	
	Student	28,308 (33)		11,370 (37)		84 (9)	
	Volunteer	4522 (5)		1519 (5)		31 (3)	
	Other: health care	5568 (6)		1755 (6)		141 (15)	
	Other:- non–health care	10,301 (12)		3561 (12)		132 (14)	
	Not specified	9354		2464		N/A^b^	
**Age (years), n (%)**							
	<20	9142 (15)		3489 (17)		15 (2)	
	20-29	27,871 (47)		9976 (47)		248 (27)	
	30-39	13,505 (23)		4542 (21)		294 (32)	
	40-49	5938 (10)		2045 (10)		198 (21)	
	50-59	2470 (4)		810 (4)		111 (12)	
	60-69	613 (1)		196 (1)		51 (6)	
	70+	357 (1)		83 (0)		7 (1)	
	Not specified	36,499		11,794		2	
**Gender, n (%)**							
	Female	29,877 (46)		9479 (42)		291 (32)	
	Male	35,444 (54)		12,921 (58)		615 (67)	
	Nonbinary/other	109 (0)		39 (0)		11 (1)	
	Not specified	30,965		10,496		9	

^a^This table compares the characteristics (n [%]) of health workers who completed the focal courses and the follow-up survey. A higher proportion of course enrollees did not specify characteristics compared with survey completers. We show the numbers not specified for each but do not include them in the percentage breakdowns. For course completion, geographic region was identified via course platform analytics; however, we were unable to identify a subset, shown as “not specified” in the table. For survey completion, the geographic region was identified through survey reports and IP addresses and was fully specified across respondents. Percentages are shown for those for whom we have data on characteristics. Other course languages include Armenian, Indonesian, Kazakh, Macedonian, Dutch, Portuguese, Russian, Albanian, Kiswahili, and Somali.

^b^N/A: not applicable.

### Survey Respondents

A number of survey respondents enrolled in more than 1 course. Nearly twice as many respondents had enrolled in the COVID-19 courses (COVID-19: General Considerations, N=607; COVID-19: Initial Approach, N=534) as compared with the other emergency health courses (Ebola: Clinical Management, N=354; Rabies and One Health, N=283). As shown in Table 2, the characteristics of the survey respondents generally reflect the characteristics of enrollees and course completers, with a few exceptions. A larger proportion of survey respondents indicated that they were from LICs (171/926, 18%). Differing from the overall enrollment and course-completer data, more than one-third of respondents indicated that they were from Sub-Saharan Africa (332/926, 36%). A larger proportion of survey respondents indicated that they were health care professionals (406/926, 44%) and employed in other health care–related professions (141/926, 15%) compared with the overall enrollee and course-completer population.

### Sharing Course Content

Overall, Table 3 shows that the vast majority of survey respondents (819/926, 88%) shared the course content. While sharing is high in all countries, respondents in LMICs (402/441, 91%) were significantly more likely to share course content than those in HICs (92/114, 81%; *P*=.001). The majority of learners who reported sharing course content indicated that they shared with 10 or fewer people (542/926, 59%). On average, learners shared content with a lower bound estimate of 8.16 (SD 14.45) people. Compared with sharing among learners in HICs who shared with an average of 6.73 (SD 12.32) people, the number of people with whom learners shared content was higher in LMICs (mean of 9.48, SD 15.76 people; *P*=.08) although not statistically significant.

**Table 3:** Sharing of course materials and information by country income classificationa.

						Total (N=926)		HIC^b^ (N=114)		UMIC^c^ (N=200)		*P* value		LMIC^d^ (N=441)				*P* value						LIC^e^ (N=171)				*P* value	
**Panel A: informal sharing (shared course materials or information).** **Sharing, n (%)**						819 (88)		92 (81)		176 (88)		.079		402 (91)				.001						149 (87)				.142	
	**Sharing, by number of recipients, n (%)**																												
			1-10 people		542 (59)		61 (54)		122 (61)		.197		253 (57)				.459						106 (62)						.156
			11-25 people		136 (15)		15 (13)		25 (13)		.867		73 (17)				.377						23 (13)						.944
			26-50 people		67 (7)		11 (10)		18 (9)		.849		30 (7)				.301						8 (5)						.100
			>50 people		74 (8)		5 (4)		11 (6)		.667		46 (10)				.047						12 (7)						.360
	Lower bound number of recipients, mean (SD)				8.16 (14.45)		6.73 (12.32)		7.13 (13.02)		.789		9.48 (15.76)				.084						6.89 (13.59)				.916		
**Panel B: Use in official trainings (used course materials in an official training). Used course in training, n (%)**						563 (61)		46 (40)		113 (56)		.006				284 (64)				<.001		120 (70)				<.001			
	**Course use in training, by number of trainees, n (%)**																												
			1-10 people		370 (40)		28 (25)		73 (37)		.029				185 (42)				.001		84 (49)				<.001				
			11-25 people		94 (10)		9 (8)		19 (10)		.633				44 (10)				.501		22 (13)				.188				
				26-50 people	49 (5)		6 (5)		11 (6)		.929				25 (6)				.867		7 (4)				.644				
				>50 people	50 (5)		3 (3)		10 (5)		.313				30 (7)				.094		7 (4)				.513				
		Lower bound number of trainees, mean (SD)			5.65 (12.54)		3.82 (10.00)		5.39 (12.28)		.247				6.46 (13.65)				.054		5.06 (11.20)				.343				

^a^This table shows the rate of informal sharing (as measured by a question asking whether learners had recommended the course or informally shared course content with other individuals) and use in an official training (as measured by a question asking whether learners had shared materials from the course as part of an official training). We also show a lower-bound estimate of sharing and number of trainees, calculated assuming learners shared with the lowest number of people in their indicated survey category response. We show differences by World Bank income classifications: HIC, UMIC, LMIC, and LIC. Tests of comparison in course sharing and adaptation by country income classification were conducted using Pearson chi-square test. We used one-way ANOVA to compare differences in the mean number of sharing recipients and trainees by country classification. We show comparisons of UMICs, LMICs, and LICs with HICs.

^b^HIC: high-income country.

^c^UMIC: upper-middle-income country.

^d^LMIC: lower-middle-income country.

^e^LIC: low-income country.

Course content was commonly used in official trainings (61%, 563/926). Use of the content in official training was significantly higher in LICs (120/171, 70%; *P*<.001), LMICs (284/441, 64%; *P*<.001), and UMICs (113/200, 56%; *P*=.006) than in HICs (46/114, 40%). The majority of the trainings in which course content was shared engaged 10 or fewer people (370/563, 67%). The lower bound estimated number of trainees was, on average, 5.65 (SD 12.54) people. Compared with the mean number of trainees in HICs (mean of 3.82, SD 10.00 people), significantly more were trained on average in LMICs (mean of 6.46, SD 13.65 people; *P*=.054).

### Characteristics of Course Sharing, Adaptation, and Accessibility

Learners shared course content widely with many in their personal and professional networks, including most notably colleagues (486/819, 59%) and friends (364/819, 44%). As shown in Table 4, this pattern was generally consistent across income country classifications. Likewise, across all income country classifications, sharing was highest among health care professionals (363/819, 44%) and learners working in other health care–related fields (126/819, 15%).

**Table 4:** Characteristics of course sharing by country income classification^a^.

		Total (N=819)	HIC^b^ (N=92)	UMIC^c^ (N=176)	*P* value	LMIC^d^ (N=402)	*P* value	LIC^e^ (N=149)	*P* value
**With whom did you share? n (%)**									
	Classmate(s) if a student	197 (24)	10 (11)	43 (24)	.008	107 (27)	.001	37 (25)	.008
	Colleague(s)	486 (59	52 (57)	106 (60)	.558	229 (57)	.938	99 (66)	.122
	Employee(s) whom you supervise	176 (21)	17 (18)	26 (15)	.433	89 (22)	.440	44 (30)	.055
	Employer	108 (13)	12 (13)	17 (10)	.397	49 (12)	.822	30 (20)	.159
	Family member(s)	220 (27)	22 (24)	39 (22)	.745	123 (31)	.204	36 (24)	.965
	Friend(s)	364 (44)	36 (39)	63 (36)	.591	186 (46)	.214	79 (53)	.036
	Patient(s) if a health care worker	180 (22)	14 (15)	35 (20)	.348	98 (24)	.058	33 (22)	.187
	Student(s) if an educator	160 (20)	9 (10)	23 (13)	.431	96 (24)	.003	32 (21)	.019
**How did you share course information? n (%)**									
	Shared course website link	489 (60)	51 (55)	102 (58)	.692	240 (60)	.453	96 (64)	.164
	Shared course files via offline technology (eg, USB, DVD, CD)	105 (13)	7 (8)	14 (8)	.920	54 (13)	.126	30 (20)	.009
	Shared course audio files on the radio	78 (10)	5 (5)	10 (6)	.933	48 (12)	.069	15 (10)	.205
	Shared course information via email	242 (30)	23 (25)	31 (18)	.152	132 (33)	.144	56 (38)	.043
	Shared course information via text message	108 (13)	9 (10)	15 (9)	.732	57 (14)	.264	27 (18)	.078
	Shared course information via WhatsApp	298 (36)	19 (21)	67 (38)	.004	165 (41)	<.001	47 (32)	.065
	Shared course information via word of mouth	290 (35)	35 (38)	62 (35)	.649	144 (36)	.689	49 (33)	.414
	Shared hard copy course materials (eg, printed handouts)	92 (11)	8 (9)	7 (4)	.111	53 (13)	.238	49 (33)	.100
**What components did you share? n (%)**									
	Videos	284 (35)	22 (24)	51 (29)	.377	156 (39)	.007	55 (37)	.035
	Audio files	17 (2)	3 (3)	9 (5)	.486	0 (0)	<.001	5 (3)	.968
	Slides	318 (39)	21 (23)	60 (34)	.057	158 (39)	.003	79 (53)	<.001
	Quizzes	189 (23)	13 (14)	29 (16)	.616	105 (26)	.015	42 (28)	.012
	Text from the course	214 (26)	20 (22)	37 (21)	.892	113 (28)	.214	44 (30)	.183
	Transcripts	133 (16)	9 (10)	13 (7)	.497	85 (21)	.012	26 (17)	.101
	Downloadable documents	263 (32)	23 (25)	45 (26)	.919	128 (32)	.199	67 (45)	.002
	Infographics	111 (14)	12 (13)	20 (11)	.687	65 (16)	.456	14 (9)	.375
	Discussions	147 (18)	13 (14)	15 (9)	.154	91 (23)	.071	28 (19)	.349
	Photographs	9 (1)	0 (0)	1 (1)	.469	3 (1)	.406	5 (3)	.076
	Other	47 (6)	4 (4)	11 (6)	.520	21 (5)	.729	11 (7)	.343
**Did you modify any content shared? n (%)**									
	Printed content to be available offline (eg, handouts)	29 (4)	4 (4)	8 (5)	.941	5 (1)	.045	12 (8)	.262
	Adapted content to distribute via text message or WhatsApp	216 (26)	7 (8)	42 (24)	.001	125 (31)	<.001	42 (28)	<.001
	Added culture or local contextual information	133 (16)	11 (12)	11 (6)	.106	80 (20)	.076	31 (21)	.079
	Added additional explanations in local language	178 (22)	18 (20)	18 (10)	.033	99 (25)	.303	43 (29)	.107
	Adjusted content to meet local guidelines /regulations	143 (17)	8 (9)	17 (10)	.797	81 (20)	.010	37 (25)	.002
	Made changes to course animations and images	73 (9)	5 (5)	8 (5)	.748	42 (10)	.139	18 (12)	.088
	Translated all or part of the course into a local language	142 (17)	10 (11)	27 (15)	.314	76 (19)	.067	29 (19)	.078
	Other modifications not described above	76 (9)	10 (11)	15 (9)	.531	33 (8)	.414	18 (12)	.776
**Did you have any difficulties when sharing? n (%)**									
	No difficulties	381 (47)	44 (48)	73 (41)	.320	187 (47)	.820	77 (52)	.561
	Web-based connectivity	178 (22)	7 (8)	29 (16)	.043	99 (25)	<.001	43 (29)	<.001
	Language barriers	120 (15)	8 (9)	18 (10)	.688	60 (15)	.118	34 (23)	.005
	Technical difficulty	77 (9)	6 (7)	8 (5)	.490	47 (12)	.148	16 (11)	.270
	Reaching intended audience was difficult	61 (7)	4 (4)	5 (3)	.516	35 (9)	.162	17 (11)	.059
	Translation difficulty	70 (9)	5 (5)	10 (6)	.933	38 (9)	.217	17 (11)	.118
	Other	41 (5)	4 (4)	8 (5)	.941	21 (5)	.729	8 (5)	.723
**What are the professions of the sharers? n (%)**									
	Government	45 (5)	3 (3)	9 (5)	.486	24 (6)	.302	9 (6)	.335
	Health care professional	363 (44)	42 (46)	85 (48)	.681	168 (42)	.499	68 (46)	.998
	Community health worker	85 (23)	7 (17)	12 (14)	.705	43 (26)	.224	23 (34)	.050
	Nurse	119 (33)	23 (55)	28 (33)	.018	51 (30)	.003	17 (25)	.002
	Paramedic /emergency medicine technician	18 (5)	4 (10)	7 (8)	.808	6 (4)	.105	1 (1)	.049
	Pharmacist	26 (7)	0 (0)	4 (5)	.153	18 (11)	.027	4 (6)	.109
	Physician	111 (31)	8 (19)	33 (39)	.025	47 (28)	.239	23 (34)	.094
	Traditional /complementary medicine	4 (1)	0 (0)	1 (1)	.480	3 (2)	.383	0 (0)	N/A^f^
	International organization (eg, WHO, and UN)	19 (2)	1 (1)	6 (3)	.258	7 (2)	.654	5 (3)	.272
	Nongovernmental organization	52 (6)	5 (5)	8 (5)	.748	24 (6)	.844	15 (10)	.205
	Student	66 (8)	2 (2)	20 (11)	.009	37 (9)	.024	7 (5)	.315
	Volunteer	31 (4)	4 (4)	5 (3)	.516	16 (4)	.872	6 (4)	.903
	Other: health care	126 (15)	18 (20)	17 (10)	.022	71 (18)	.668	20 (13)	.204
	Other: non–health care	117 (14)	17 (18)	26 (15)	.433	55 (14)	.240	19 (13)	.226

^a^This table shows the characteristics of course sharing by World Bank income classifications: HIC, UMIC, LMIC, and LIC. Tests of comparison in course sharing and adaptation by country income classification were conducted using Pearson chi-square test. We show comparisons of UMICs, LMICs, and LICs with HICs.

^b^HIC: high-income country.

^c^UMIC: upper-middle-income country.

^d^LMIC: lower-middle-income country.

^e^LIC: low-income country.

^f^N/A: not applicable.

Across income country classifications, learners were most likely to share course content via website links (489/819, 60%), followed by WhatsApp (298/819, 36%), word of mouth (290/819, 35%), and email (242/819, 30%). Sharing course information via WhatsApp was highest in UMICs (67/176, 38%; *P*=.004) and LMICs (165/402, 41%; *P*<.001) and significantly higher than in HICs (19/92, 21%). Learners in LICs were more likely to share course files via offline technology (30/149, 20%; *P*=.009) than those in HICs (7/92, 8%). The course components shared by learners also varied by country income classification. Learners in LICs or LMICs were more likely to share videos (LMICs, *P*=.007; LICs, *P*=.04), slides (LMICs, *P*=.003; LICs, *P*<.001), quizzes (LMICs, *P*=.02; LICs, *P*=.01), transcripts (LMICs, *P*=.01), and downloadable documents (LICs, *P*=.002) than those learners in HICs.

Learners adapted and made modifications to the MOOCs to share their content with others in all countries, with a few notable variations across income country classification. Over a quarter of learners (216/819, 26%) adapted course content to make it easier to distribute via text message or WhatsApp. This type of adaptation was significantly more likely in UMICs (42/176, 24%; *P*=.001), LMICs (125/402, 31%; *P*<.001), and LICs (42/149, 28%; *P*<.001) than in HICs (7/92, 8%). Compared with learners in HICs, learners in LICs or LMICs were also more likely to make adaptations to add cultural or contextual information (LMICs, *P*=.076; LICs, *P*=.079), to adjust content to meet local guidelines and regulations (LMICs, *P*=.010; LICs, *P*=.002), and to address linguistic barriers by translating all or part of the course into a local language (LMICs, *P*=.067; LICs, *P*=.078).

These adaptations may have been made to address accessibility challenges. Internet connectivity was the biggest challenge to accessing and sharing MOOCs for over a quarter of all learners in LMICs (99/402, 25%; *P*<.001) and LICs (43/149, 29%; *P*<.001) and significantly more learners experienced internet connectivity challenges than learners in HICs (7/92, 8%). In addition, nearly a quarter of learners in LICs (34/149, 23%; *P*=.005) expressed that language barriers presented a challenge to accessing and sharing content, which was significantly higher than the proportion expressing this challenge in HICs (8/92, 9%). Even so, a quarter of learners in LICs pointing to language barriers is relatively low considering the levels of literacy of the common course languages (eg, English), suggesting that courses are not reaching a broader audience in LICs.

Across all survey respondents, learners in UMICs, LMICs, and LICs were more likely to express that they found the OpenWHO MOOC content less accessible than learners in HICs (Table 5). The vast majority of learners in HICs (89/114, 78%) expressed that the MOOC content was accessible to most people in their country, compared with just more than half of learners in UMICs (110/200, 55%; *P*<.001) and LMICs (229/441, 52%; *P*<.001). Less than a third of learners in LICs (53/171, 31%; *P*<.001) expressed that MOOC content was accessible to most people in their country. This pattern holds for accessibility among patients, as indicated by health care professionals, with accessibility particularly low in LICs where 38% (54/171) of learners indicated the MOOC content is accessible to only very few people.

**Table 5:** Accessibility of OpenWHO courses by country income classification^a^.

Accessibility of OpenWHO		HIC^b^ (N=114)	UMIC^c^ (N=200)	*P* value	LMIC^d^ (N=441)	*P* value	LIC^e^ (N=171)	*P* value
**In your country, n (%)**								
	Accessible to most	89 (78)	110 (55)	<.001	229 (52)	<.001	53 (31)	<.001
	Accessible to some	19 (17)	60 (30)	.009	124 (28)	.013	61 (36)	<.001
	Accessible to very few	6 (5)	30 (15)	.009	88 (20)	<.001	57 (33)	<.001
**In your personal network, n (%)**								
	Accessible to most	80 (70)	103 (52)	.001	208 (47)	<.001	45 (26)	<.001
	Accessible to some	24 (21)	57 (29)	.147	139 (32)	.029	63 (37)	.005
	Accessible to very few	10 (9)	40 (20)	.009	94 (21)	.002	63 (37)	<.001
**In your professional network, n (%)**								
	Accessible to most	81 (79)	123 (66)	.022	232 (56)	<.001	53 (34)	<.001
	Accessible to some	16 (16)	50 (27)	.029	124 (30)	.003	71 (45)	<.001
	Accessible to very few	6 (6)	14 (7)	.593	57 (14)	.027	34 (22)	.001
**Among your patients, n (%)**								
	Accessible to most	55 (65)	69 (42)	.001	175 (45)	.001	32 (22)	<.001
	Accessible to some	15 (18)	56 (34)	.007	128 (33)	.006	58 (40)	<.001
	Accessible to very few	14 (17)	38 (23)	.225	85 (22)	.285	54 (38)	.001

^a^This table shows responses to questions about the accessibility of OpenWHO courses by World Bank income classifications: HIC, UMIC, LMIC, and LIC. Tests of comparison in course sharing and adaptation by country income classification were conducted using Pearson chi-square test. We show comparisons of UMICs, LMICs, and LICs with HICs.

^b^HIC: high-income country.

^c^UMIC: upper-middle-income country.

^d^LMIC: lower-middle-income country.

^e^LIC: low-income country.

## Discussion

### Principal Findings

This study is one of the first to examine the sharing and adaptation of health MOOCs globally. The WHO expedites the production of learning materials as soon as evidence-based science becomes available for any disease outbreak to ensure that communities and health workers have resources that are reliable, readily accessible, and language appropriate [3]. Given this diverse scope and the ongoing challenges of accessing web-based platforms and MOOCs in different countries, we sought to understand how these 4 MOOCs are reaching a global audience. Overall, we found that OpenWHO MOOC content is highly shared across all regions of the world, presenting a tremendous opportunity for the dissemination and scaling of health information. However, adaptation (ie, contextualization and localization) is critical to improve reach in LMICs or LICs.

This study demonstrates that in addition to the official course enrollers and completers, the OpenWHO course content is reaching an even larger audience than that which is observable from platform analytics. The majority of survey respondents indicated that they shared course content or used course content in official training. They were most likely to share videos, share content via WhatsApp, and adapt content to share using technologies such as WhatsApp or text message. While respondents across all country classifications shared course content and materials, learners in LMICs were most likely to share course content. On average, they also reached the largest number of people with their sharing.

The variation in the ways MOOCs are being shared and adapted, as well as the reported accessibility challenges in LICs or LMICs, suggests that MOOCs designed purely for an individual learner in an online format will not optimize knowledge and skill dissemination or most effectively reach geographies with the lowest level of resources. To access this content, learners must rely on others with the access, means, and expertise to adapt and translate the MOOCs to overcome technical limitations. Our findings are consistent with previous research on the OpenWHO platform indicating that although the platform is designed for low-bandwidth use, such as providing downloadable materials, technical limitations remain [16]. In LMICs or LICs, we observed a greater level of effort to distribute content in offline ways to address technical difficulties, such as adapting content to distribute via text message and WhatsApp and by sharing course files via offline technology (eg, USB, DVD, and CD). The lower rate of adaptation in HICs may reflect that the content, made by course developers in HICs, requires less modification to share. It may also reflect that there are more widely available alternative sources of content in HICs. These findings present a tremendous opportunity for course developers to devise new ways of designing and disseminating courses that could have a dramatic scaling effect, particularly in areas with more access challenges.

In addition to addressing technical limitations, we observed that learners in LMICs or LICs were significantly more likely to modify content to address local guidelines, cultural contexts, and languages. Learners in LICs, in particular, expressed that language barriers created an accessibility challenge for content. Our findings reflect the literature that argues that to be relevant, MOOCs need to be adaptable and linguistically and culturally responsive [3,41,46,47]. Successful content adaptations of this nature likewise require substantial time, resources, and particular linguistic and medical expertise, all of which form barriers to individual learners who wish to adapt and share courses in order to spread knowledge and strengthen the local health care response. These findings require that we intentionally design MOOCs that facilitate and support adaptation and sharing by local health professionals to address needs in LMICs or LICs where the content is critical to promoting information equity during public health emergencies.

### Limitations and Future Research

Our study findings are limited to the population who responded to our survey. As a result, our findings do not represent the entire population that accessed the OpenWHO platform or enrolled in the MOOCs and shared content but did not respond to the survey. Our survey respondents are learners with email addresses and the time, interest, and capacity to respond to an online survey. Respondents were also those with the inclination and ability to respond to a survey in English, French, or Spanish. That only a quarter of survey respondents in LICs indicated experiencing language barriers—relatively low considering the levels of literacy of the common course languages, (eg, English, French, and Spanish) overall in LICs—could suggest that MOOCs are not reaching a broader audience in LICs but also likely reflects a limitation with the selected survey response. Furthermore, our findings omit feedback from offline learners exposed to course content through the sharing we describe in this study. We hence cannot speak to the quality of the learning experience among such learners, who would be able to shed light on ways to improve the creation of MOOC content to better facilitate alternative content distribution paths.

Future research must illuminate how best to develop tools and provide the additional supports needed for learners and institutions wishing to adapt and share MOOC content. In addition, it is important that we evaluate the impact of shared trainings on “secondary” learners’ knowledge, skills, and behaviors. Such investigations are essential to ensure that shifts toward online and digital educational approaches during health emergencies do not exacerbate global gaps in access to health care and health knowledge.

### Conclusions

There exists a strong demand for OpenWHO MOOCs health emergencies trainings as demonstrated by the high rates of sharing materials across countries of all income levels. Local content sharing presents a tremendous opportunity for organizations and governments to exponentially scale the impact of MOOCs. MOOC developers must design courses for easier distribution via commonly identified formats for sharing such as text message and WhatsApp. Learners in LMICs or LICs were more likely to adapt content to meet local cultural, contextual, and linguistic needs but faced increased barriers to adapting and sharing materials. This study advances our understanding of how to design and disseminate educational materials to promote equitable access to critical health information during times of crisis.

## Appendix

Multimedia Appendix 1 Learner survey in English.

## Abbreviations

HIC: high-income country
LIC: low-income country
LMIC: lower-middle-income country
MOOC: massive open access online course
UMIC: upper-middle-income country
WHO: World Health Organization

## Data Availability

Some data are available on reasonable request to the corresponding author.
