# Supplementary material for: Global Use, Adaptation, and Sharing of Massive Open Online Courses for Emergency Health on the OpenWHO Platform: Survey Study
Source: J Med Internet Res. 2025 Jan 10;27:e52591. doi: 10.2196/52591 (PMC11759914; doi:10.2196/52591)
Supplement: Multimedia Appendix 1 [file jmir_v27i1e52591_app1.pdf]

Supplement 1. Learner Survey in English

**Section A: Course Information**		
A.1	When did you enroll in the course?	[drop down: month / years]
A.2	Did you complete the course?	( ) yes<br>( ) no
A.3	How long did it take you to complete the course?	( ) < 2 weeks<br>( ) 3-6 weeks<br>( ) > 7 weeks
A.4	What was the main reason you enrolled in the course? Select one.	( ) Earn the certificate of completion<br>( ) Fulfill an education requirement<br>( ) Improve my professional skills<br>( ) Personal learning (unrelated to profession)<br>( ) Recommended by my employer / institution<br>( ) Required by my employer / institution
A.5	How did you first hear about the course?	( ) Colleague<br>( ) Employer<br>( ) Friend<br>( ) Internet search<br>( ) OpenWHO email<br>( ) Professional association<br>( ) School<br>( ) Social media<br>( ) WHO website<br>( ) Other
A.6	If you selected “other” to the previous question, please specify where you heard about the course.	
A.7	Did you enroll in other course(s)? If so, which ones did you enroll in? Select all that apply.	( )<br>( ) Other<br>( ) None

Section B: Course Sharing		
B.1	Did you **recommend the course or informally share course content** (i.e.- informally sharing includes sharing materials or information in social media, emails, or other communications) with any of the following individuals? Select all that apply.	[ ] Classmate(s) if a student<br>[ ] Colleague(s)<br>[ ] Employee(s) whom you supervise<br>[ ] Employer<br>[ ] Family member(s)<br>[ ] Friend(s)<br>[ ] Patient(s) if a healthcare worker<br>[ ] Student(s) if an educator<br>[ ] None of the above
B.2	How did you tell others about the course and/or share information from the course? Select all that apply.	[ ] Shared the course website link<br>[ ] Shared the course through offline digital technology/hardware (i.e., USB, DVD, CD, etc.)<br>[ ] Shared the course audio files on the radio<br>[ ] Shared information from the course via email<br>[ ] Shared information from the course via SMS<br>[ ] Shared information from the course via WhatsApp<br>[ ] Shared information from the course informally via word of mouth<br>[ ] Share printed hard copy materials from the course (e.g., printed handouts)<br>[ ] Not applicable - did not recommend or share course information
B.3	With how many individuals did you recommend or informally share course information?	[drop down, None, 1-10, 11-25, 26-50 and 50+]
B.4	Did you share materials from the course as part of an **official training at your institution** with any of the following? Select all that apply.	[ ] Classmate(s) if a student<br>[ ] Colleague(s)<br>[ ] Employee(s) whom you supervise<br>[ ] Employer<br>[ ] Family member(s)<br>[ ] Friend(s)<br>[ ] Patient(s) if a healthcare worker<br>[ ] Student(s) if an educator<br>[ ] None of the above

B.5	How was the official training offered? Select one.	( ) Fully in person<br>( ) Fully online<br>( ) Partially online, partially in person<br>( ) Not applicable - did not share materials from course as part of an official training
B.6	With how many individuals did you share course materials through an **official** training or educational experience at your institution?	[drop down, None, 1-10, 11-25, 26-50 and 50+]
B.7	If you did share the course content with anyone, what aspects of the curriculum did you decide to share? Select all that apply.	[ ] Video<br>[ ] Audio<br>[ ] Slides<br>[ ] Quizzes<br>[ ] Text pages<br>[ ] Transcripts<br>[ ] Downloadable documents (such as WHO technical guidance)<br>[ ] Infographics / posters<br>[ ] Discussions<br>[ ] Photos/ images/ illustrations<br>[ ] Other<br>[ ] Not applicable- did not share course content
B.8	If you decided to share the course content, why did you decide to share the curriculum? Select all that apply.	[ ] My community/ patients/ colleagues/ etc. needed the information<br>[ ] I found the materials easy to understand<br>[ ] I found the materials easy to share<br>[ ] I was tasked with teaching/ training others on the content<br>[ ] Other<br>[ ] Not applicable- did not share course content
B.9	When sharing course information, did you **modify the content** in any of the following ways? Select all that apply.	[ ] Printed content to be available offline (e.g. handouts, pamphlets, posters)<br>[ ] Adapted content to distribute via SMS or WhatsApp<br>[ ] Added cultural or local contextual information<br>[ ] Added additional explanations in local language<br>[ ] Adjusted content to meet local guidelines / regulations (e.g. from Ministry of Health)<br>[ ] Made changes to course animations and images<br>[ ] Translated all or part of the course into a local language<br>[ ] Other<br>[ ] Not applicable - did not share course information
B.10	If you decided to share and/ or modify content, what difficulties did you face when sharing the content? Select all that apply.	[ ] I did not face any difficulties<br>[ ] I had technical difficulties with internet connectivity and wifi when attempting to share the content<br>[ ] There were language barriers with the content for the audiences I wanted to share with

		[ ] I faced difficulties while making technical changes (i.e- the platform was difficult to work with, I didn't have the technical knowledge to make the changes I wanted to make, etc.)<br>[ ] Reaching the intended audience was difficult<br>[ ] Accessing the materials was difficult<br>[ ] I had difficulty translating the materials<br>[ ] Other<br>[ ] Not applicable - did not share course information
B. 11	If you did share the course content with anyone, could you tell us more about the experience? Why did you share?	[open text]
**Section C: Learner Characteristics**		
C.1	In what country do you reside?	[drop down]
C.2	What is your preferred language to acquire knowledge?	( ) Arabic<br>( ) Chinese<br>( ) English<br>( ) French<br>( ) Portuguese<br>( ) Russian<br>( ) Spanish<br>( ) Other
C.3	If you selected "other" to the previous question, please specify your preferred language.	[free text]
C.4	Is the language you selected in C2 the same as your native language?	( ) Yes<br>( ) No
C.5	In your **country**, how accessible are OpenWHO digital education materials?	( ) accessible to most<br>( ) accessible to some<br>( ) accessible to very few
C.6	Think about your **personal network** (e.g., family and friends). How accessible are OpenWHO digital education materials?	( ) accessible to most<br>( ) accessible to some<br>( ) accessible to very few

C.7	Think about your **professional network** (e.g., colleagues and others in your field of work). How accessible are OpenWHO education materials?	( ) accessible to most<br>( ) accessible to some<br>( ) accessible to very few<br>( ) not applicable - not currently working in a professional capacity
C.8	If you are a healthcare provider, how accessible are OpenWHO digital educational materials to your **patients or clients**?	( ) accessible to most<br>( ) accessible to some<br>( ) accessible to very few<br>( ) not applicable - not a healthcare provider
C.9	What best describes your current profession or affiliation? Select one.	( ) Community health worker<br>( ) National ministry of health<br>( ) Non-governmental organization (NGO) or non-profit organization<br>( ) Nurse/ Nurse midwife/ Nursing assistant<br>( ) Paramedic or emergency medicine technician<br>( ) Pharmacist<br>( ) Physician<br>( ) Physician assistant or nurse practitioner<br>( ) Professor / teacher<br>( ) Public health practitioner (epidemiology, biostatistics, etc.)<br>( ) Researcher<br>( ) Student<br>( ) Traditional or complementary medicine practitioner<br>( ) Unemployed / not currently employed<br>( ) UN country team or UN partner organization<br>( ) Volunteer<br>( ) WHO consultant or affiliate<br>( ) Other healthcare not listed above<br>( ) Other non-healthcare not listed above
C.10	In what type of setting do you usually work?	( ) Higher education institution (university, college)<br>( ) Hospital<br>( ) Ministry of health (public health)<br>( ) Other healthcare setting<br>( ) Other education setting<br>( ) Other not listed above<br>( ) Not applicable
C.11	What best describes your employer?	( ) For-profit company<br>( ) Government (e.g. Ministry of Health)<br>( ) Higher education institution (e.g. university, college)<br>( ) Intergovernmental organization (e.g., UN, UN partner organization)<br>( ) Non-governmental organization (NGO) / Non profit organization

		( ) School (primary or secondary level)<br>( ) Other<br>( ) Not applicable
C.12	Do you supervise anyone in your current role?	( ) Yes<br>( ) No<br>( ) Not applicable
C.13	How old are you?	( ) <18 years<br>( ) 18-29 years<br>( ) 30-39 years<br>( ) 40-49 years<br>( ) 50-59 years<br>( ) 60-69 years<br>( ) 70 years or older<br>( ) Prefer not to answer
C.14	What best describes your gender?	( ) Female<br>( ) Male<br>( ) Transgender Female<br>( ) Transgender Male<br>( ) Gender variant/ non conforming<br>( ) Prefer to self describe as [open text]<br>( ) Prefer not to answer
C.15	Can we contact you to learn more about your experience with the course? If yes, please share your contact information. (Optional)	Name: [open text]<br>Email: [open text]<br>Phone: [open text]
C.16	In which of the following WHO courses did you enroll? Select all that apply.	[ ] COVID-19 General Considerations<br>[ ] COVID-10 Acutely Ill Patients<br>[ ] Ebola Clinical Management<br>[ ] Rabies and One Health
